# Jatropha Oil Derived Sophorolipids: Production and Characterization as Laundry Detergent Additive

**DOI:** 10.1155/2013/169797

**Published:** 2013-12-21

**Authors:** Kasturi Joshi-Navare, Poonam Khanvilkar, Asmita Prabhune

**Affiliations:** Biochemical Sciences Division, National Chemical Laboratory, Homi Bhabha Road, Pune, Maharashtra 411008, India

## Abstract

Sophorolipids (SLs) are glycolipidic biosurfactants suitable for various biological and physicochemical applications. The nonedible Jatropha oil has been checked as the alternative raw material for SL synthesis using *C. bombicola* (ATCC22214). This is useful towards lowering the SL production cost. Through optimization of fermentation parameters and use of resting cell method, the yield 15.25 g/L could be achieved for Jatropha oil derived SL (SLJO) with 1% v/v oil feeding. The synthesized SL displayed good surfactant property. It reduced the surface tension of distilled water from 70.7 mN/m to 33.5 mN/m with the Critical Micelle Concentration (CMC) value of 9.5 mg/L. Keeping the prospective use of the SL in mind, the physicochemical properties were checked along with emulsion stability under temperature, pH stress, and in hard water. Also antibacterial action and stain removal capability in comparison with commercial detergent was demonstrated. SLJO enhanced the detergent performance. Based on the results, it can be said that SLs have utility as fabric cleaner with advantageous properties such as skin friendly nature, antibacterial action, and biodegradability. Therefore SLs are potential green molecules to replace synthetic surfactants in detergents so as to reduce harm caused to environment through detergent usage.

## 1. Introduction

In terms of production volume, surfactants belong to the most important classes of industrial chemicals with a current total world production exceeding 13 million tonnes per year [1]. About half that volume is used in household and laundry detergents and the other half in a wide variety of industrial sectors, particularly the chemical, textile, food, and paper industry, cosmetics, personal, and health care, agriculture, and so forth. The majority of the currently used surfactants are petroleum-based and are produced by chemical means. These compounds are often toxic to the environment, and their use may lead to significant ecological problems, particularly in washing applications as these surfactants inevitably end up in the environment after use [2]. The ecotoxicity, bioaccumulation, and biodegradability of surfactants are therefore issues of increasing concern. Phosphates are being recognized as one of the essential nutrients contributing to the eutrophication and detergents are one of the many sources of phosphate discharged to the environment [3]. Therefore attempts should be made to reduce the detergent load in to the environment. In this scenario the biosurfactants are promising alternatives to synthetic surfactants as they can be produced from renewable feedstock by natural fermentation process. Also they are readily biodegradable and display low ecotoxicity [1].

Sophorolipids (SLs) are a kind of microbial extracellular biosurfactants produced by nonpathogenic yeasts, such as *C. bombicola, Candida apicola,* and *Candida bogoriensis *[4]. They are generally present in the form of disaccharide sophoroses (2-O-*β*-D-glucopyranosyl-D-glucopyranose) linked *β* glycosidically to the hydroxyl group at the penultimate carbon of fatty acids. When SLs are solved in water, they lower the surface tension from 72.8 mN/m down to 40 to 30 mN/m, with a critical micelle concentration of 40 to 100 mg/L. The hydrophilic/lipophilic balance is 10 to 13, making SLs useful as detergents or as stabilizers for oil-in-water emulsions [2]. Thus this amphiphilic molecule, SL, satisfies above mentioned criteria and stands as an ideal candidate to replace chemical surfactants in detergents.

Economy is often the bottleneck of biotechnological processes. Biosurfactants have to compete with surfactants of petrochemical origin in three aspects: cost, functionality, and production capacity (Makkar and Cameotra 2002). The success of biosurfactant production depends on the development of cheaper processes and the use of low cost raw materials, which account for 10–30% of the overall cost [5]. Therefore attempts are being made to explore cheap and renewable sources for SL production. Deshpande and Daniels produced 120 g/L SL using animal fat from meat processing industry (100 g/L precursor feeding) [6]. Daniel et al. used whey concentrate and rapeseed oil for SL production (yield obtained was 422 g/L with 100 g/L oil feeding) [7]. Ashby et al. synthesized 60 g/L SL from Biodiesel coproduct stream (100 g/L precursor feeding) [8]. Fleurackers produced 49 g/L SL using waste frying oil (37.5 g/L oil feeding) [9]. Shah et al. reported utilization of restaurant waste oil as a precursor for SL production (yield obtained was 34 g/L with 40 g/L oil feeding) [10]. Daverey and Pakshirajan produced 63.7 g/L SLs using low cost media based on sugarcane molasses and soybean oil, sunflower oil, or olive oil with oil feeding 100 g/L [11]. Also cheese whey, molasses, corn steep liquor, and residues from vegetable oil refinery have been reported as raw material for SL production [5].

Here we have explored the utility of the nonedible Jatropha oil to be used as raw material for SL synthesis. The oil is nonedible owing to the presence of antinutritional factors such as phorbol esters [12]. Due to the unfavourable odour, colour, and composition, the uses of Jatropha oil are limited to some extent. However it is majorly composed of saturated or unsaturated fatty acids with chain lengths of 16 or 18 carbon atoms, making it an ideal substrate for direct incorporation and the consequent high SL production and yield. During SL synthesis, the vast majority of fatty acids are either elongated or metabolized to C16 or C18 fatty acids by the *C. bombicola* ATCC22214. The best yields are obtained for oleic acid (C18:1) [2]. Jatropha oil contains up to 80–95% of such fatty acids [12]. Refer to Table 1 for fatty acid composition. The Jatropha seed contains 46.27% oil. It has been reported that the toxicity and disagreeable odour of seed are due to protein which accounts for 22.50% w/w of seed. 4.56% w/w total ash content of seeds indicates presence of abrasive solids, soluble metallic soaps, and silica residue in seed. The typical SL producing yeast strain *C. bombicola* (ATCC22214) is a robust organism and it can survive and produce SL in presence of the alkaloids in Jatropha oil. There is a single recent report on the use of the nonedible oils including for SL production [13]. The authors reported the yield value of SLJO as 6.0 g/L with 10% w/v oil feeding.

Owing to the amphiphilic nature of SLs, their applicability as an ingredient of laundry detergent has been identified previously [14, 15] but still not in wide scale practice as a commodity product with the exception of few SL based cleaners by some multinational companies (current SLs producers include Belgium-based Ecover, France-based Groupe Soliance, Japan-based Saraya, and Republic Korea-based MG Intobio with current production scale reported to be small) and there is room for SL based detergents with better process economics. Hence here an attempt has been made to produce a biological surfactant using Jatropha oil as an economical substrate. The actual data evaluating the washing performance of SL or combination of SL with commercial detergent formulation is being reported here. Thus applicability of SL to be used in detergent formulations as an alternative to harmful chemical surfactants has been shown.

## 2. Experimental Procedures

### 2.1. Microorganisms and Their Maintenance

Nonpathogenic yeast, *C. bombicola* (ATCC22214), was used for the production of SL. It was maintained on MGYP (malt extract: 0.3 g%, glucose: 2 g%, yeast extract: 0.3 g%, peptone: 0.5 g%, and agar: 2.0 g%) slants. The microorganism was subcultured in every 4 weeks and maintained at 4°C in a refrigerator [16]. *Staphylococcus aureus* (ATCC29737) and *Escherichia coli* (ATCC8739) were procured from National Collection of Industrial Microorganisms (NCL). The cultures were maintained on nutrient agar slants. The microorganisms were subcultured in every 4 weeks and maintained at 4°C in a refrigerator till required further.

### 2.2. Chemicals and Reagents

All chemicals and solvents used in this study were of analytical grade and supplied by either Himedia Pvt. Ltd., India, or Merck India Ltd. Jatropha oil was purchased from local market in Pune, India, in a single batch.

### 2.3. SL Yield Maximization

#### 2.3.1. Effect of Media Constituents

In addition to primary and secondary carbon sources, media contain nitrogen source, growth factors, buffer components, and other minerals which show significant effect on SL yields. Different media previously reported for maximum production of SL were tried. The media compositions have been listed in Table 2 [16–20].

Seed culture was prepared by inoculating 5 mL of respective media with *C. bombicola* (ATCC22214), followed by incubation at 28°C, 180 rpm for 24 h. This seed culture was transferred to 45 mL of fresh medium along with 0.5 mL of Jatropha oil dispersed in 0.5 mL of absolute ethanol. Incubation was continued further. The samples were withdrawn after 96 and 120 h for SL estimation.

The SL was harvested by the procedure previously reported by Shah and Prabhune 2007. Culture medium was centrifuged at 5,000 rpm, at 10°C for 20 minutes. The cell pellet was washed with ethyl acetate to recover the SLs precipitated during centrifugation. The supernatant was extracted twice with equal volumes of ethyl acetate, organic layer was dried over anhydrous Na_2_SO_4_, and the solvent was removed by rotary evaporation. The yellowish brown semicrystalline product was washed twice with n-hexane and yields were determined [20].

#### 2.3.2. Effect of Primary, Secondary Carbon Feed Concentration

The medium and incubation period giving maximum yield were fixed and the glucose concentrations were varied, namely, 5, 7 and 10% w/v. In case of secondary carbon source, oil feeding was varied within the range 1 to 5% v/v.

#### 2.3.3. Effect of Physical Parameters

Optimal temperature for *C. bombicola *ATCC22214 is reported to be 28.8°C (information from the National Collection of Yeast Cultures, UK) [2]. To check the effect of incubation temperature, the fermentations were carried out at different temperatures namely, 28, 31, and 33°C. To check the effect of initial pH on SL production, the initial pH values were adjusted approximately to 4.0, 5.0, 6.0, and 7.0 using 0.1 M HCl/NaOH.

The fermentation, extraction procedures were essentially done the same as mentioned before. The experiments were carried out in triplicates in 250 mL Erlenmeyer flasks containing 50 mL of the production media.

#### 2.3.4. Production of SL by Resting Cell Method

SL is known to be the stationery phase metabolite. Hence in order to reduce the carbon consumption for biomass increase and cell maintenance purpose and direct it majorly towards SL production, the cells were pregrown in optimum medium, that is, medium F which also gave maximum biomass production and then subjected to the SL production medium containing the precursors for SL production, that is, glucose and Jatropha oil as per the optimized conditions.

Seed culture of *C. bombicola* ATCC22214 was inoculated in 10 mL of freshly prepared medium F (malt extract 0.3 g%, yeast extract 0.3 g%, glucose 5 g%, and mycological peptone 0.5 g%) and incubated for 24 h at 28°C under shaking condition (180 rpm). This preinoculum was added to 90 mL of medium F in a 500 mL Erlenmeyer flask and incubated for 48 h at 28°C on a shaker (180 rpm). Cells were harvested by centrifugation and washed twice with glass distilled water under sterile conditions. The cell pellets (biomass ~15 g dry weight per liter of medium) were redispersed in simple production medium which is the solution of 10% glucose with 1 mL of Jatropha oil (dispersed in 1 mL of ethanol) and the flask was kept on a shaker 180 rpm at 28°C for 96 h. As a result of interaction of the yeast biomass with glucose and fatty acid, a brown and viscous liquid (crude SL) could be seen settled at the bottom of the flask which was separated using a pipette tip cut at nozzle and subjected to extraction procedure as mentioned in earlier part of experimental section [13, 21, 22]. The cells were separated from the broth by centrifugation at 5000 rpm, 10°C for 20 minutes. Broth supernatant was also subjected to extraction procedure in order to recover the un-settled SLs. The separated cells were again dispersed in simple production medium supplemented with oil to produce SL. This way the biomass can be exploited for up to 3 times with satisfactory yield of SL.

### 2.4. Characterization of the Synthesized SL

After the yield maximization experiments, the MALDI-MS (matrix assisted laser desorption/ionization-mass spectrometry), NMR analysis was performed to know about structural composition of the SL sample. Further the surface active properties of SLJO were evaluated.

#### 2.4.1. Structural Characterization of SLJO

*(a) MALDI-MS Analysis of SL.* SLJO sample 1 mg was dissolved in 1 mL of methanol. Further 5 *μ*L of SLJO sample was mixed with 20 *μ*L of dithranol matrix and MALDI-MS study was done on AB SCIEX TOF/TOF 5800.

*(b) SL Structure Confirmation with* ^*1*^ *H NMR.* Two milligrams of SLJO sample was dissolved in 0.5 mL of deuterated chloroform. ^1^H NMR (200 MHz) spectra were recorded by Bruker AC200 at 25°C. Chemical shift was expressed in ppm. Tetramethylsilane was used as an internal standard.

#### 2.4.2. Minimum Surface Tension and Critical Micelle Concentration (CMC)

Minimum surface tension and critical micelle concentration of SLJO were estimated using a KRUSS surface tensiometer K11 by Wilhelmy plate method.

Stock of SLJO was prepared in MilliQ water (pH = 7.0) and diluted appropriately to get different concentrations. The concentration range used was 0.95–850 mg/L. A clean, dry 100 mL glass beaker was filled with the different concentrations of SLJO solution and subjected to surface tension measurement one by one. The beaker was placed on the sample platform of the Kruss K11 tensiometer. The platinum surface tension probe was removed from the tensiometer hook and rinsed with deionized water and dried with the blue part of the flame from the propane torch. The probe was then air-cooled and reinserted onto the tensiometer hook. The surface tensions of the solutions of different concentrations were measured as described in the tensiometer operating manual. All surface tension measurements were the average of 4 readings recorded at an interval of 30 seconds.

To determine critical micelle concentration (CMC), the surface tension was measured as a function of surfactant concentration. Surface tension was then plotted versus log surfactant concentration. The resulting curve had a nearly horizontal portion at concentrations higher than the CMC and had a negative steep slope at concentrations less than the CMC. The CMC was calculated as that concentration of the curve where the flat portion and the extrapolated steep slope intersected. The surface tension beyond CMC was the value in the flat portion of the curve.

#### 2.4.3. Emulsification Activity and Stability

Emulsification activity and stability of the SLJO were tested with oleic acid as an organic solvent using a modified method of Cirigliano and Carman [23, 24]. SLJO stock was prepared in double distilled water pH 7.0 (stock strength = 0.5 mg/mL). 1 mL of SLJO stock was mixed with 1 mL of the oil substrate (oleic acid). Then, mixture was shaken vigorously in a vortex mixer for 2 minutes and allowed to sit for 10 minutes before measuring its absorbance at 600 nm. Emulsification activity was expressed as the absorbance of the mixture at 600 nm (*A*_600_). The readings were noted in duplicate and the average values were noted down. The stability of the resulting emulsion was expressed as the decay constant (*k*_*d*_) estimated from the following linear relationship between absorbance (*A*_600_) and time (days) as represented in the following equation:

(1) $$\begin{array}{c} \log A_{600}=-k_{d}\times t. \end{array}$$

#### 2.4.4. Effect of Environmental Parameters on Emulsifying Property

The effect of environmental parameters such as water hardness, pH, and temperature on emulsification activity was determined by varying the levels of the individual parameters one at a time by keeping the other parameters at a fixed level. The modified method of Daverey and Pakshirajan 2010 has been used [24].

*(a) Effect of Water Hardness on Emulsifying Property.* To study the effect of water hardness, simulated hard water was used. The simulated hard water was prepared as follows: stock of 10 mg/mL was prepared by mixing 5 mg/mL of each calcium chloride (CaCl_2_·2H_2_O) and magnesium sulfate (Mg(SO_4_)·7H_2_O). Then stock was diluted using double distilled water to prepare moderately hard water (0.12 mg/mL) and hard water (0.18 mg/mL). Therefore the resultant moderately hard water contains 1.08 mM of Ca^2+^ and 1.0 mM of Mg^2+^ while hard water contains 1.6 mM of Ca^2+^ and 1.5 mM of Mg^2+^.

To test the effect of hardness on emulsification activity and stability of SLJO, 1 mL SL solution (0.5 mg/mL) was prepared in moderately hard water and hard water and incubated for 1 hour at 30°C and then emulsification activity and stability were assessed according to the procedure mentioned earlier.

*(b) Effect of pH on Emulsifying Property.* For testing the effect of pH on emulsification activity and stability of SLJO, 1 mL SL (0.5 mg/mL) solution was prepared in 0.2 M acetate/phosphate buffer solutions (for detailed buffer preparation see Supplementary Material available online at http://dx.doi.org/10.1155/2013/169797) having various pH values, namely, 4.0, 5.0, 6.0, 7.0, and 8.0, and incubated for 1 hour at 30°C and emulsification activity and stability were checked.

*(c) Effect of Temperature on Emulsifying Property.* For observing the effect of temperature, 1 mL SLJO (0.5 mg/mL) solution was incubated for 30 minutes at various temperatures in the range 20–80°C and emulsification activity and stability was then evaluated using oleic acid as the substrate according to the procedure mentioned earlier.

#### 2.4.5. Evaluation of Antibacterial Property of SL

*Staphylococcus aureus* (ATCC29737) and *Escherichia coli* (ATCC8739) were used as the test organisms, representatives of Gram positive and Gram negative genera.

Protocol was followed to check the bacterial inhibition by SLJO. Appropriate dilution of bacterial cell suspension was exposed to different SL concentrations (50–500 *μ*g/mL) and mixtures were incubated at 28°C for 4 h at 180 rpm. Then 50 *μ*L of mixture was spread plated on nutrient agar plates and incubated at room temperature for 24 h and colonies were counted. Experiments were done in triplicate and the average values were noted.

### 2.5. Evaluation of Surfactant Properties of SLJO

In view of their intended use as detergent additive, wetting property and contact angle reduction were examined.

*(a) Examining the Wetting Property*. Wetting property or wettability was measured by canvas disc method (Wilham et al., 1973). The test was carried out by measuring the sinking time of canvas disk [25]. The wetting property of SLJO, SDS (sodium dodecyl sulphate), and Triton X-100 (octyl phenol ethoxylate) was evaluated. 100 mL test solutions of concentration 0.01 g%, 0.1 g%, and 1 g% were prepared in double distilled water, pH 7.0. Canvas disc of 1-inch diameter was placed in Gooche funnel and it was then inverted in a beaker containing test solution. Sinking time, that is, time required for the canvas disc to sink to the bottom of beaker, was measured.

*(b) Wetting Property of SL in Combination with Synthetic Surfactants.* It is well known that certain mixtures of surfactants can provide better performance than pure surfactants for a wide variety of applications [26]. Wetting property of individual surfactants as well as combinations of SLJO with synthetic surfactants SDS and Triton X-100 was checked since we aim to use the SL as supplements in detergents. Stock solution of SL (0.01 g%) and chemical surfactants (0.01 g%) were prepared. SL and chemical surfactants were mixed in three ratios, 1 : 3 (i.e., 25 mL of SL solution was mixed with 75 mL of synthetic surfactant solution), 1 : 1, and 3 : 1. Further their wetting properties were assessed.

#### 2.5.1. Determination of Contact Angle

Contact angle measurements were performed using Goniometer: G-10 contact angle meter. SL stock solution of 1 *μ*g/mL was prepared in double distilled water. 10 *μ*L volume was dropped onto of the test surface. Three different surfaces, namely, glass, teflon and stainless steel, have been tried out. And *θ*_*C*_ values were measured.

### 2.6. Comparative Performance Assessment of SL with Synthetic Detergent Using Detergency Test

In detergency test, the comparative performance of SLJO, a commercial detergent preparation, and SLJO in combination with commercial detergent has been evaluated against 4 different stains, namely, coffee, turmeric, oil, and poster color, on 2 different types of fabrics, namely, cotton and polyester.

Following method was practiced. Pieces of 2 × 2 inches were cut of cotton and polyester cloth. Cloth pieces were placed on saran wrap and stained, another piece of saran wrap was placed on it, and heavy weight was put on it for 5–10 minutes and then stains were allowed to dry overnight. Next day, stained pieces of cloth were soaked individually in 0.1 g% solution of SLJO and 0.1 g% solution of commercial detergent for 10 minutes. Soaked pieces of cloth were hand-washed for approximately 1-2 minutes. Excess water from cloth was squeezed out and cloth pieces were allowed to dry normally and results were visually noted [27, 28].

Same procedure was carried out to test the stain removal capacity of SL in combination with commercial detergent, in 1 : 1 proportion with appropriate controls.

## 3. Results and Discussion

### 3.1. SL Yield Maximization

In order to maximize the product yield, 6 media differing in the proportion of sugar, nitrogen source, presence of buffer components, and so forth were chosen. For media compositions, refer to Section 2.1, Table 2. Out of the 6 media used, medium F was found to give maximum yield of SLJO. Also it was seen that yield value decreased after 96 h of incubation. The set of optimized parameters for the production of SLJO are temperature 28°C, pH 4.0, glucose concentration 10 g%, and fatty acid precursor volume 1%. (refer to electronic supplementary material for comparative values).

SL yield has been improved through the use of optimized parameters combined with resting cell method. SL production is associated with stationary phase of growth. Therefore the cells which were already used for synthesizing SLs could give satisfactory yields when supplemented with just glucose and Jatropha oil. 15.25 g/L of SL derived from Jatropha oil could be obtained with 1% v/v oil feeding. Resting cell method allowed the use of same biomass for up to 3 times, thus making the process still more efficient. With the second and third time use of the cells, 15.1 g/L and 13.25 g/L of SL yield were obtained, respectively. After that SL production dropped considerably.

### 3.2. Characterization of SLJO

**Figure 1:**
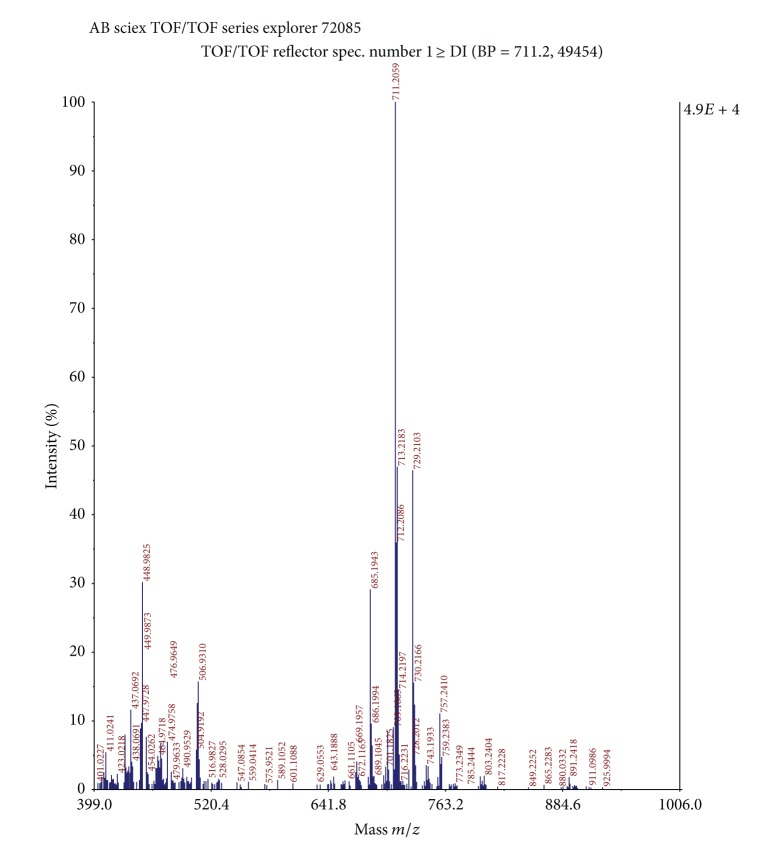
MALDI/MS spectrum of the SLJO preparation.

Typical structure of SLs consists of a sophorose (dimeric sugar) linked *β*-glycosidically to terminally or subterminally hydroxylated fatty acid with chain lengths 16–18 [2]. SL occurs as a mixture of compounds differing in their acetylation, lactonization, and position of hydroxylation. Therefore a pure precursor fatty acid leads to formation of different forms of SL in crude product. As starting material, that is, Jatropha oil in present case, is a mixture of fatty acids, multiple forms of SL molecules with varied hydrophobic moieties in addition to above-mentioned criteria are expected in product. This was confirmed with the MALDI-MS analysis of the sample. Prominent peaks from the mass spectrum were correlated to sodium adducts [M^+^ + H^+^ + Na^+^] of the expected forms of SLs. Different forms of SLs derived from palmitic (*m/z* 685, 703), stearic (*m/z* 647, 713), oleic (*m/z* 669, 687, 711, 729), linoleic (*m/z* 709, 727), and trace amounts of arachidic (*m/z* 716, 759) acid were detected. In the synthesized SL, diacetylated SL of C18:1, that is, oleic acid *m/z* 711, was detected as the most abundant structural form. Constituent structural forms corresponding to the observed *m/z* values have been listed in Table 3 along with their relative proportions (refer to Figure 1 for mass spectra). It can be observed that ~57% of the SL consists of oleic acid as the hydrophobic moiety. The approximate percent fractions of palmitic, stearic linoleic, and arachidic acid are 13.5, 17, 9, and 2, respectively. Hence correlation with Jatropha oil composition could be established except for C18 fatty acids. It can be said that probably the organism is capable of introducing saturation.

The ^1^H NMR spectrum of the SLJO preparation was assigned to a typical glycolipid-type structure. And characteristic proton chemical shift peaks could be observed. Protons of (–CH_3_) of fatty acid resonated at 1.20. Protons of (–CH_2_) bonded to carboxylic group of fatty acid resonated at 1.99. Resonance of protons belonging to sophorose moiety resulted in peaks within the region 4–4.5. Appearance of peaks around 5.3 was attributed to the signals from (–CH=CH–), that is, unsaturation in the fatty acid chain. The data was found to be in agreement with previously reported SL-NMR data from relevant references [13, 29].

Before proceeding to further analysis, surfactant property of the synthesized SL was qualitatively confirmed by oil displacement test. The surface active properties were checked using different experimental and analytical methods.

#### 3.2.1. Minimum Surface Tension and Critical Micelle Concentration (CMC)

**Figure 2:**
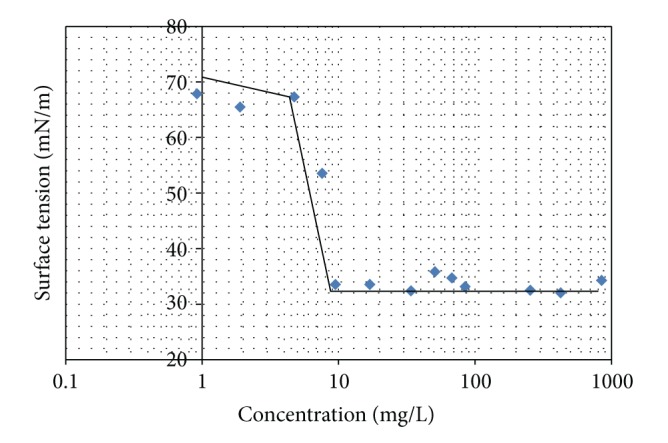
Minimum surface tension and critical micelle concentration of SLJO.

It was seen that SLJO reduced the surface tension of distilled water from 70.714 mN/m to 33.512 mN/m at the CMC value of 9.5 mg/L as depicted in Figure 2 (refer to supplementary information for different SLJO concentrations and corresponding surface tension values). Typically the CMC values of SLs fall within the range 40–100 mg/L [2]. Daverey and Pakshirajan have mentioned that the SL produced using low cost fermentative medium could lower the surface tension to 34.15 mN/m and the CMC value was observed to be 59.43 mg/L. The crude SL preparations may show CMC values as high as 150 mg/L [11]. Thus it is worth to mention that SLJO is showing a low CMC value. On the other hand, SDS, a common synthetic surfactant, lowers the surface tension of double distilled water up to 25 mN/m with the CMC value 2.24 g/L (0.008 M) [30]. Another common surfactant Triton X-100 shows the CMC value ~150 mg/L (0.22–0.24 mM) and lowers the surface tension of double distilled water up to 32 mN/m [31].

#### 3.2.2. Emulsification Activity and Stability

The emulsification activity and stability of SLJO and synthetic surfactants, namely, SDS and Triton X-100, have been noted in Table 4. Emulsification activity and stability of SLJO were observed to be better than those of standard chemical surfactant, Triton X-100, whereas emulsification activity of SLJO was less than that of SDS but stability of emulsion was better than that of SDS. The trend observed for stability of emulsion was SLJO > Triton X-100 > SDS.

#### 3.2.3. Effect of Environmental Parameters on Emulsifying Property

As an ingredient of detergent, SLJO has to perform satisfactorily in extreme physical conditions and different water qualities. Therefore the effect of different parameters on emulsifying property was explored.

*(a) Effect of Water Hardness on Emulsifying Property.* Results mentioned in Table 5 imply that emulsification index as well as stability of emulsion gets affected by the hardness of water. Stability of emulsions formed by SLJO was better than that of Triton X-100. Stability of emulsions formed by SLJO and Triton X-100 decreased in hard water. Stability of emulsion formed by SDS in distilled water was very low as compared to SL and Triton X-100, but it was observed to behave erroneously in hard water. 

*(b) Effect of pH on Emulsifying Property. *pH is known to be one of the most important environmental factors influencing the performance of any biosurfactant. pH alters the net charge on surfactant molecule and thus its orientation at the interface.

The experiment was repeated twice and the emulsification indices were calculated from the average *A*_600_ values. It was observed that emulsification activity of SLJO was comparable between pH values of 5.0–8.0 while the emulsification index value at pH 4.0 was 6 times low as compared to other pH values. Classically detergent formulations make the pH alkaline. SLJO was found to retain the surfactant activity at alkaline pH which makes SL suitable for combination with commercial detergents.

It was observed that emulsions formed by SLJO were stable within the pH range of 5.0–8.0. The decay constant of SLJO is −11.3184 at pH 4.0 (refer to supplementary information for details). The results are in agreement with those of the report by Daverey and Pakshirajan, 2009, wherein they have used sugarcane molasses and soybean oil for SL synthesis and found that emulsifying activity was maximum at pH 7.0 and highest stability at pH 8.0 [32].

*(c) Effect of Temperature on Emulsifying Property.* It was observed from Table 6 that, for the SLJO, emulsifying property is best at 60°C and then goes on decreasing. Probably temperature rise enhances micellarization and beyond 60°C destruction occurs. When the emulsion stability was assessed, it was found that the stability was best at 20°C and then it decreased with increasing temperature. Hence the energy costs in washing processes can be lowered. Daverey and Pakshirajan, 2009, reported that the SL synthesized using sugarcane molasses and soybean oil showed low emulsification activity at 20°C and beyond that emulsification activity increased but stability decreased [32].

#### 3.2.4. Evaluation of Antibacterial Property of SLJO

SLs are known to possess antimicrobial properties [2]. The proposed primary mechanism of action of these surfactants is membrane lipid order perturbation, which compromises the viability of microorganisms [33].

**Figure 3:**
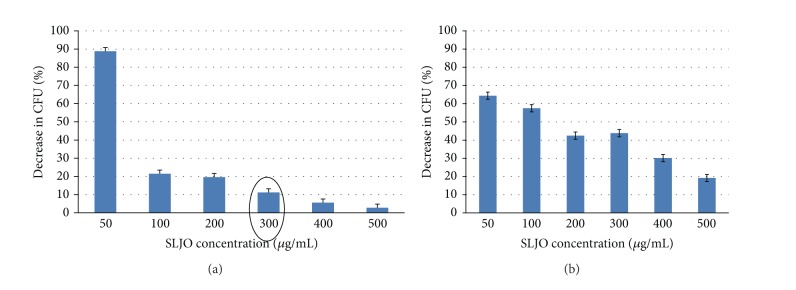
Antibacterial action of SLJO (a) against *Staphylococcus aureus *and (b) against *Escherichia coli*. The MIC_90_ value has been marked with circle.

The minimum inhibitory concentration required to inhibit 90% of the organisms;S that is, MIC_90_ values were determined. Against *Staphylococcus aureus, *MIC_90_ of SLJO was 300 *μ*g/mL, while against *Escherichia coli* SLJO could not achieve 90% inhibition till 500 *μ*g/mL. Thus it can be said that SLJO is more active against gram positive index bacterium. The antibacterial character of SLJO is an additional advantage while using it in detergent formulations (refer to Figure 3).

### 3.3. Evaluation of Surfactant Properties of SLJO

#### 3.3.1. Determination of Contact Angle

The contact angle value is dictated by the interaction between surfactant molecule and the solid surface. SLJO was able to improve spreading and reduce the contact angle. SLJO brought down the value on teflon (95° to 56°) and stainless steel (85° to 42°). No much change in contact angle was seen in case of glass surface. Thus water droplets will spread evenly on teflon and stainless steel to give low *θ*_*C*_ value, while on glass higher *θ*_*C*_ values approaching 90° or even more are expected. This suggested suitability of the SLJO for hard surface cleaning and degreasing applications.

#### 3.3.2. Examining the Wetting Property

Dose dependent wetting performance of SL was assessed using canvas disc method. At 0.01% concentration of SL, 8.8 minutes were required for sinking, while at 1 g% concentration sinking time decreased to 1.15 minutes. In the first phase of washing, textile fibres and soil must be wetted as thoroughly as possible by the wash liquor. Wetting is a complex process which is determined by the interaction of the different interfacial tensions between the solid surface, the liquid, and the gas phase. A contact angle *θ* between the solid and a drop of a liquid applied to its surface is formed and this can be taken as a measure of wetting [34]. From the results of contact angle determination and wetting, it can be expected that SLJO will reduce the soaking time required during washing process, thus making the interface for stain removal available within shorter period.

#### 3.3.3. Wetting Property of SLs in Combination with Synthetic Surfactants

**Figure 4:**
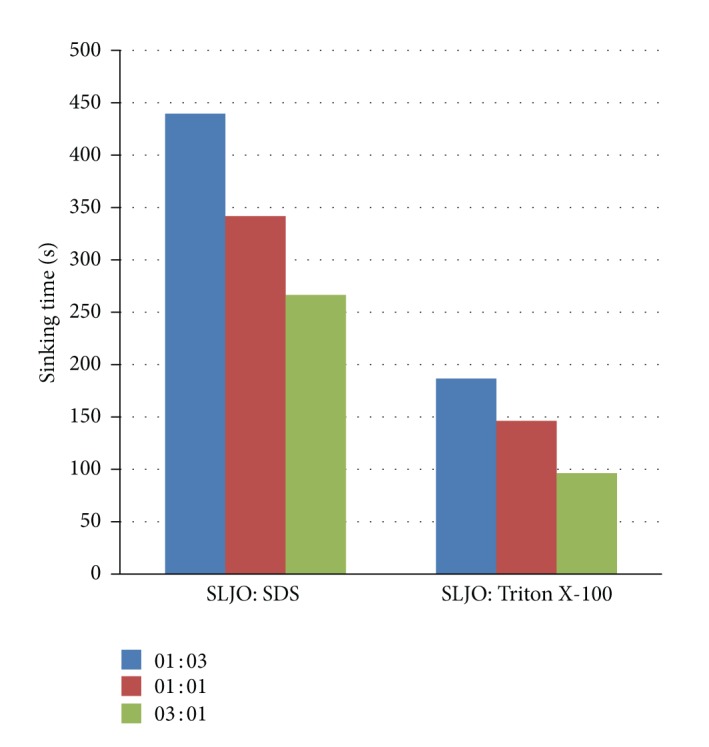
Effect of SLJO addition to improve wetting property of synthetic surfactants. Wetting improved with increasing proportion of SLJO.

Sinking time required in case of combinations of SLJO with SDS and Triton X-100 was less than the time required for individual SLJO, SDS, and Triton X-100 (refer to Figure 4). With increased proportion of SL in combination, sinking time decreased which means that SLJO enhances the wetting property of SDS and Triton X-100. Lowest sinking time was observed for 3 : 1 combinations of SLs with synthetic surfactants (refer to supplementary information for wetting property of individual surfactants). Sinking time of canvas disk in 0.01% SDS solution was noted as 751 seconds while its combination with SLJO (1 : 3) resulted in sinking time 266 seconds. In case of Triton X-100, individual sinking time reduced from 387 seconds to 96 seconds when mixed with 3 parts of SLJO solution.

### 3.4. Comparative Performance Assessment of SLs with Synthetic Detergent Using Detergency Test

**Figure 5:**
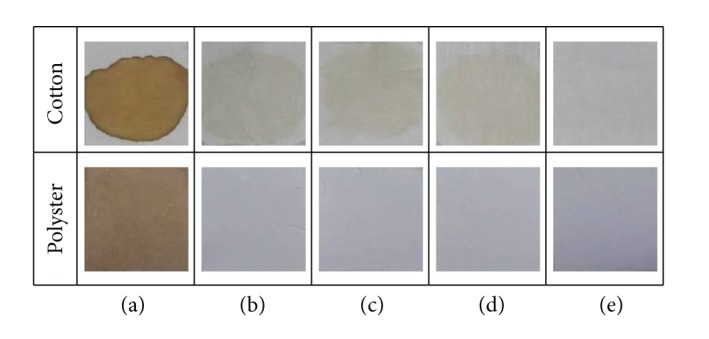
Detergency test results-cleaning performances of SLJO and commercial detergent and their combination against coffee stain. (a) Coffee stained fabrics (b) washed with commercial detergent, (c) washed with SLJO, and (d) washed with SLJO and commercial detergent 1 : 1 (e) unstained fabric.

Figure 5 is the representative of detergency test results demonstrating comparative washing performances of SLJO, commercial detergent, and the SLJO-detergent combination against coffee stain. It can be observed from Figure 5 that, with polyester fabric, total stain removal was achieved with all the combinations. With cotton, total coffee stain removal was not achieved with commercial detergent or SLJO, but SLJO-detergent combination resulted in better stain removal. Therefore it can be said that performance of SLJO nearly equals that of detergent.

Similar results were observed in case of remaining 3 types of stains, turmeric, oil, and poster color (refer to electronic supplementary material). In cases where total cleaning was not achieved with the prescribed washing protocol, visibly SLJO performance almost matched that of the commercial detergent. And the combination SLJO-detergent worked best.

In the detergency test, stains differing in their chemical nature have been used which are considered to be notorious such as caffeic acid, a yellow solid containing phenolic, acrylic group in coffee stain, and curcuminoids in turmeric. Conventionally bleach or acids are used for these kinds of tough stains which damage the fabric; on the other hand, SLs are skin friendly.

Hence the results can be summed up as there is an indication that, for majority of stains, SLJO can work as good as detergent. And through standardization of the washing procedures, there can be improvement. When combined with detergent, SLJO enhances their action. This way, reduction in the detergent load to half is really attractive and will have big positive impact.

## 4. Conclusion

In the present paper SL, a type of biosurfactants, has been produced using nonedible Jatropha oil derived from seeds of *Jatropha curcas*. The present report can be regarded as one of the first reports with reference to utilization of nonedible oil for SL production. The yield value observed was 15.25 g/L with optimized conditions and 1% v/v oil feeding. The Jatropha oil contains alkaloids and phenolics. Thus detoxification has been achieved by exploiting the robust organism, *C. bombicola*, and a valuable molecule has been produced from renewable stock. Resting cell method has been used in contrast to growth associated SL production which improved production economics.

The SLJO was found to work at low CMC value, that is, 9.5 mg/L. Other desirable properties of surfactants to work as good detergent such as wetting property, contact angle reduction, antibacterial action, and so forth have also been confirmed with the SLJO. Emulsification property of SLJO has been evaluated with special reference to changes in environmental parameters pertaining to different water qualities. Also we are reporting the use of SL for cleaning the fabric stains in comparison with commercial detergent formulation. SL acts as efficient stain cleaner. When used with detergent, it showed improved performance, thus reducing the load and harm caused to environment. This is specifically advantageous as the half-life of detergent can be up to 16 days which is detrimental to aquatic life and badly affects the ecological balance of water bodies. On the contrary, the biosurfactant SLs are biodegradable, ecofriendly, and nontoxic.

## Supplementary Material

The supplementary information contains detailed data on SL yield maximization i.e. response to variation in media composition and fermentation parameters as described in section 2.3, NMR pattern of SLJO, detailed surface tension reduction behaviour of various concentrations of SLJO. These are followed by details about emulsifying activity assessment, contact angle reduction and results of wetting property assessment of individual surfactants. The results of detergency test against remaining 3 types of stains namely turmeric, oil and poster color has been also included.

## Figures and Tables

**Table 1:** Fatty acid composition of Jatropha oil (adapted from [12]).

Fatty acid	Weight % in Jatropha oil
Palmitic acid (C16:0)	16.69
Stearic acid (C18:0)	7.67
Oleic acid (C18:1)	40.39
Linoleic acid (C18:2)	33.09
Linolenic acid (C18:3)	0.28

**Table 2:** Compositions of different media used during SL yield maximization experiments.

	Medium A [17]	Medium B [18]	Medium C [19]	Medium D [20]	Medium E [17]	Medium F [16]
Glucose	100 g/L	100 g/L	100 g/L	150 g/L	100 g/L	50 g/L
Yeast extract	5 g/L	1 g/L	1 g/L	4 g/L	—	3 g/L
Peptone	—	—	—	—	5 g/L	5 g/L
Magnesium sulphate	5 g/L	0.3 g/L	0.7 g/L	0.3 g/L	5 g/L	—
Dipotassium hydrogen phosphate	—	—	0.16 g/L	—	—	—
Potassium dihydrogen phosphate	1 g/L	—	1 g/L	6 g/L	1 g/L	—
Disodium hydrogen phosphate	—	2 g/L	—	2 g/L	—	—
Sodium dihydrogen phosphate	—	7 g/L	—	—	—	—
Sodium citrate	—	—	5 g/L	—	—	—
Sodium chloride	0.1 g/L	—	0.5 g/L	—	0.1 g/L	—
Ammonium sulphate	—	1 g/L	—	—	—	—
Ammonium nitrate	0.05 mol	—	—	—	0.05 mol	—
Ammonium chloride	—	—	1.5 g/L	—	—	—
Urea	—	—	—	2 g/L	—	—
Calcium chloride	0.1 g/L	—	0.27 g/L	—	0.1 g/L	
Malt Extract	—	—	—	—	—	3 g/L

**Table 3:** Comparative data on structural composition of SLJO.

SL structural forms	Mol. Wt.	*m*/*z* [M^+^ + H^+^ + Na^+^]	SLJO	
			Relative abundance	Approximate % composition
Nonacetylated SL of C18:0, acidic form	623	647	1.86	0.67
Monoacetylated SL of C18:1, lactonic form	645	669	6.75	2.45
Diacetylated SL of C16:0, lactonic form	661	685	29.1	10.54
Monoacetylated SL of C18:1, acidic form	663	687	6.41	2.32
Diacetylated SL of C16:0, acidic form	679	703	8.47	3.07
Diacetylated SL of C18:2, lactonic form	685	709	9.04	3.27
Diacetylated SL of C18:1, lactonic form	687	711	100	36.22
Diacetylated SL of C18:0, lactonic form	689	713	46.95	17.00
Monoacetylated SL of C20:0, acidic form	692	716	1.19	0.43
Diacetylated SL of C18:2, acidic form	703	727	15.21	5.51
Diacetylated SL of C18:1, acidic form	705	729	46.41	16.81
Diacetylated SL of 20:0, acidic form	735	759	4.73	1.71

**Table 4:** Emulsification activity and stability of SLJO and synthetic surfactants.

	Emulsification activity (*A*_600_)	Decay constant (*k*_*d*_) per day
SLJO	1.9725	−1.3824
Triton X-100	0.789	−1.8432
SDS	2.250	−5.7312

**Table 5:** Effect of water hardness on emulsifying property and stability of SLJO and Triton X-100.

Hardness	SLJO		Triton X-100	
	Emulsification activity (*A*_600_)	Decay constant (*K*_*d*_)	Emulsification activity (*A*_600_)	Decay constant (*K*_*d*_)
Distilled water	1.9725	−1.3824	0.789	−1.8432
Moderately hard water	1.846	−1.8432	1.0665	−3.6288
Hard water	0.779	−3.0528	1.531	−3.312

**Table 6:** Effect of temperature on emulsification activity and stability of SLJO.

Temperature (°C)	Emulsification activity (*A*_600_)	Decay constant (*k*_*d*_)
20	1.902	−0.9216
40	2.010	−3.2256
60	2.249	−4.1472
80	1.807	−6.3072

## Abbreviations

SL: Sophorolipid
SLJO: Jatropha oil derived sophorolipid
SDS: Sodium dodecyl sulphate
CMC: Critical micelle concentration.
