# Supplementary material for: Jatropha Oil Derived Sophorolipids: Production and Characterization as Laundry Detergent Additive
Source: Biochem Res Int. 2013 Dec 21;2013:169797. doi: 10.1155/2013/169797 (PMC3880730; doi:10.1155/2013/169797)
Supplement: Supplementary file 1 — The supplementary information contains detailed data on SL yield maximization i.e. response to variation in media composition and fermentation parameters as described in section 2.3, NMR pattern of SLJO, detailed surface tension reduction behaviour of various concentrations of SLJO. These are followed by details about emulsifying activity assessment, contact angle reduction and results of wetting property assessment of individual surfactants. The results of detergency test against remaining 3 types of stains namely turmeric, oil and poster color has been also included. [file 169797.f1.pdf]

Optimization of media and fermentation parameters

Effect of different media on yields of SLJO

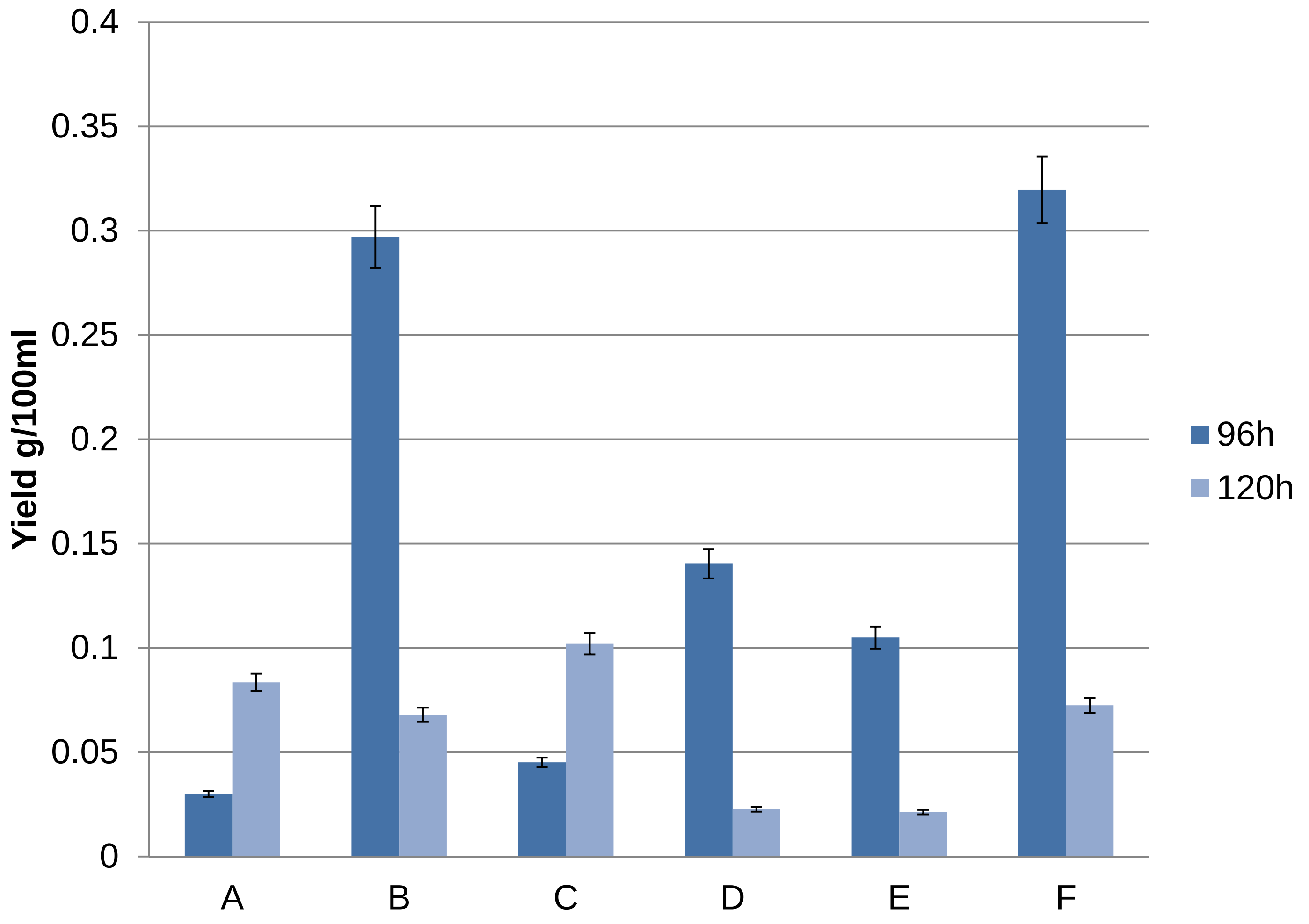

Effect of glucose concentration on SL yield

Glucose concentration (grams/100ml)	Yield (grams/100ml)
5	0.1004
7	0.1085
10	**0.1537**

Effect of precursor volume on SL yield

Precursor volume (ml/100ml)	Yield (grams/100ml) SLJO
1	**0.1336**
3	0.0868
5	0.0484

Effect of incubation temperature on SL yield

Temperature of incubation	Yield (grams/100ml)
28˚C	**0.1433**
31˚C	0.0375
33˚C	0.0101

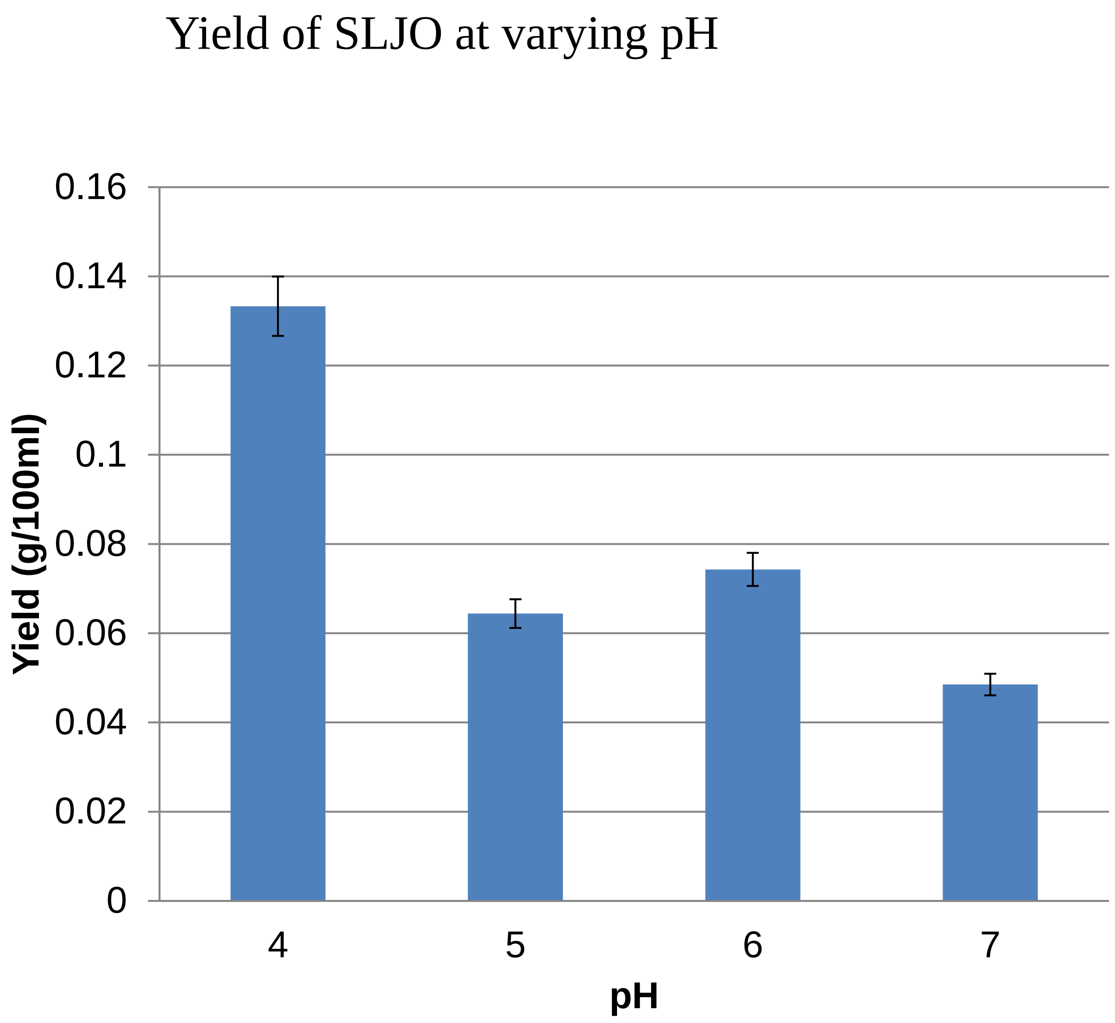

Yield of SLJO at varying pH
Yield (g/100ml)
0.16
0.14
0.12
0.1
0.08
0.06
0.04
0.02
0
4
5
6
7
pH

NMR pattern- SLJO (Sophorolipid of Jatropha oil)

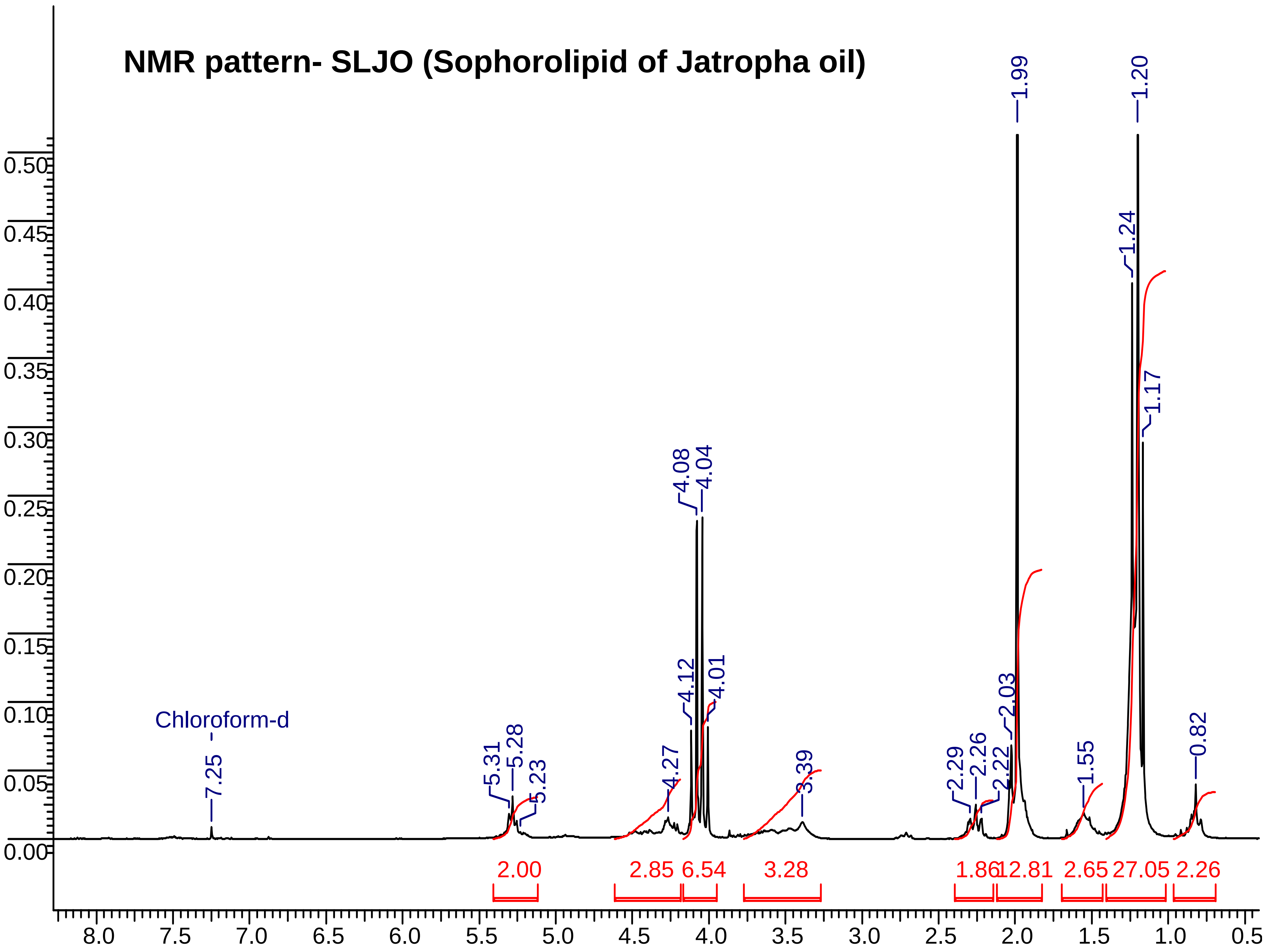

Surface tension reduction by SLJO)

Concentration (mg/l)	Surface tension (mN/m)
850	34.274
425	32.628
255	33.085
85	33.146
68	34.701
51	35.83
34	32.872
17	33.512
9.5	33.512
7.6	53.546
4.75	67.268
1.9	65.469
0.95	67.848

Effect of pH on emulsifying activity

Preparation of buffers :

Stocks used:

A: Acetic acid (0.2M)

B: Sodium acetate (anhydrous) (0.2M)

C: Sodium di-hydrogen phosphate ($NaH_2PO_4.2H_2O$) (0.2M)

D: Di-sodium hydrogen phosphate (anhydrous) (0.2M)

Additions of stocks were done as per the table given above and diluted to 50ml.

Acetate buffers		
pH	ml of A	ml of B
4.0	20.5	4.5
5.0	7.4	17.6
Phosphate buffers		
pH	ml of C	ml of D
6.0	21.9	3.1
7.0	9.7	15.2
8.0	1.3	23.7

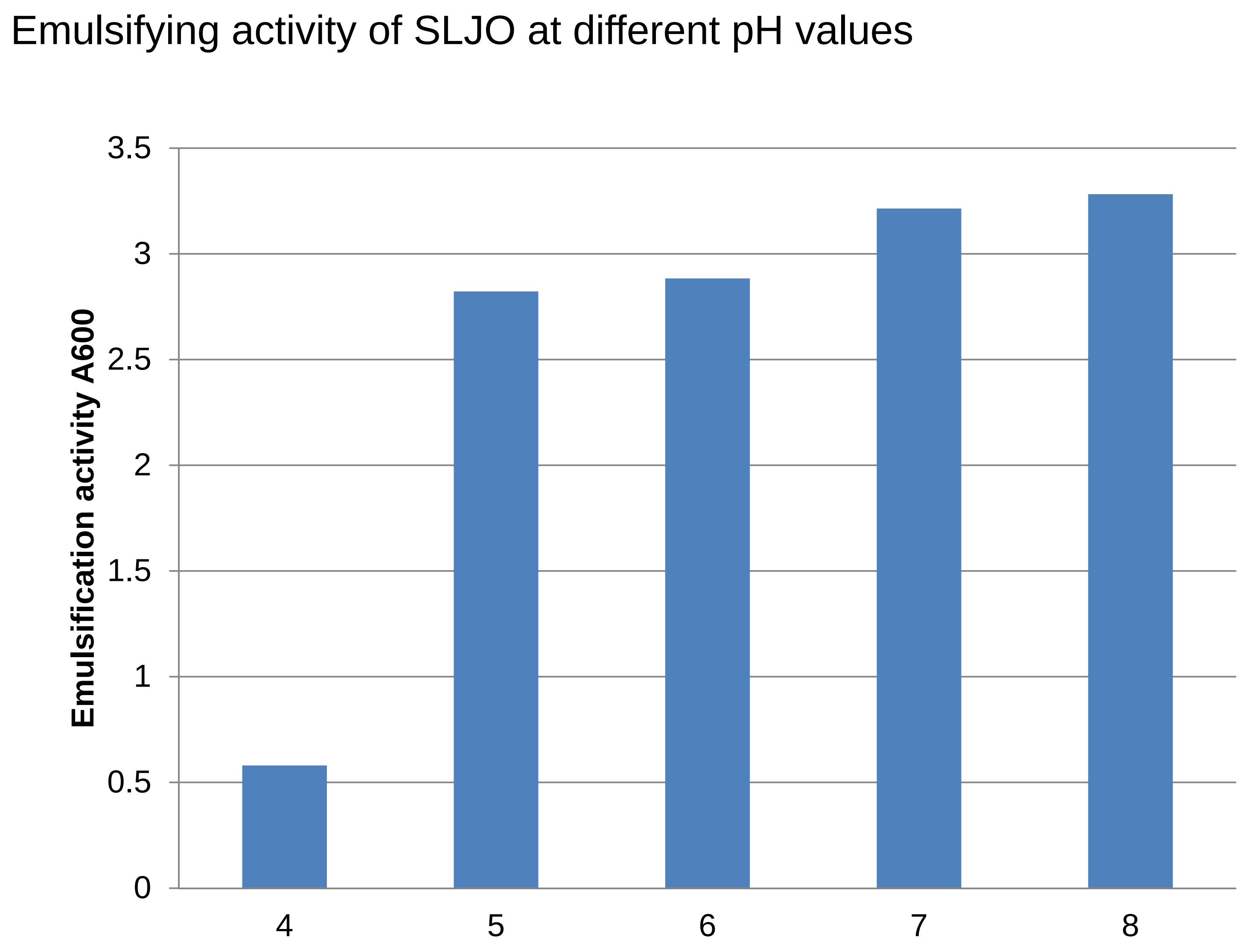
Emulsifying activity of SLJO at different pH values
Emulsification activity A600
3.5
3
2.5
2
1.5
1
0.5
0
4
5
6
7
8

Stability of SLJO emulsions at different pH values with respect to time

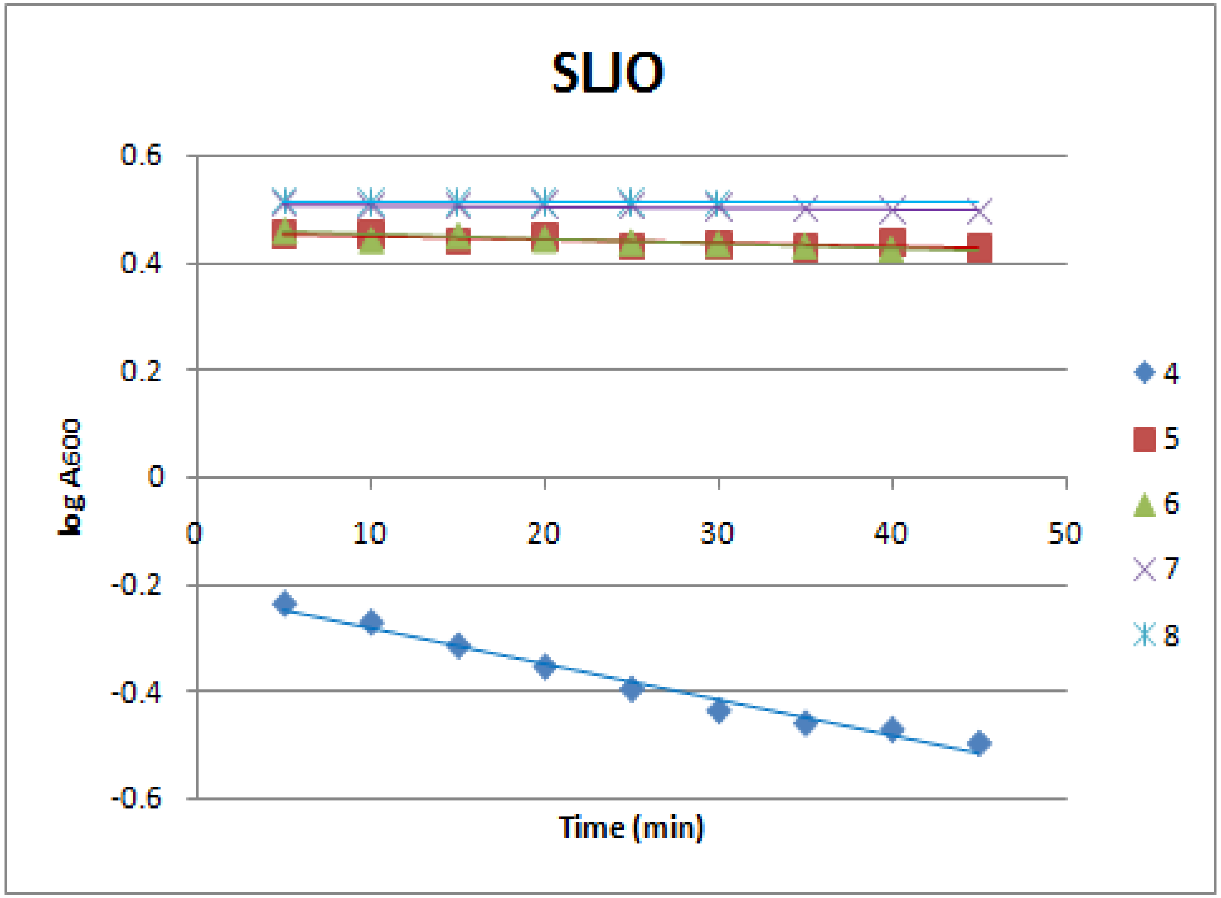

Change in contact angle of water on addition of SLJO, SLPO with typical surfaces

Sample	Contact angle value		
	Glass	Teflon	Stainless steel
Control (distilled water)	37°	95°	85°
SLJO	38°	56°	42°

Wetting property assessment- sinking time of canvas disc in different surfactant solutions

Surfactant concentration	Sinking time (in seconds)		
	SDS	Triton X-100	SLJO
0.01g%	751.16	387.27	528.07

Detergency test results

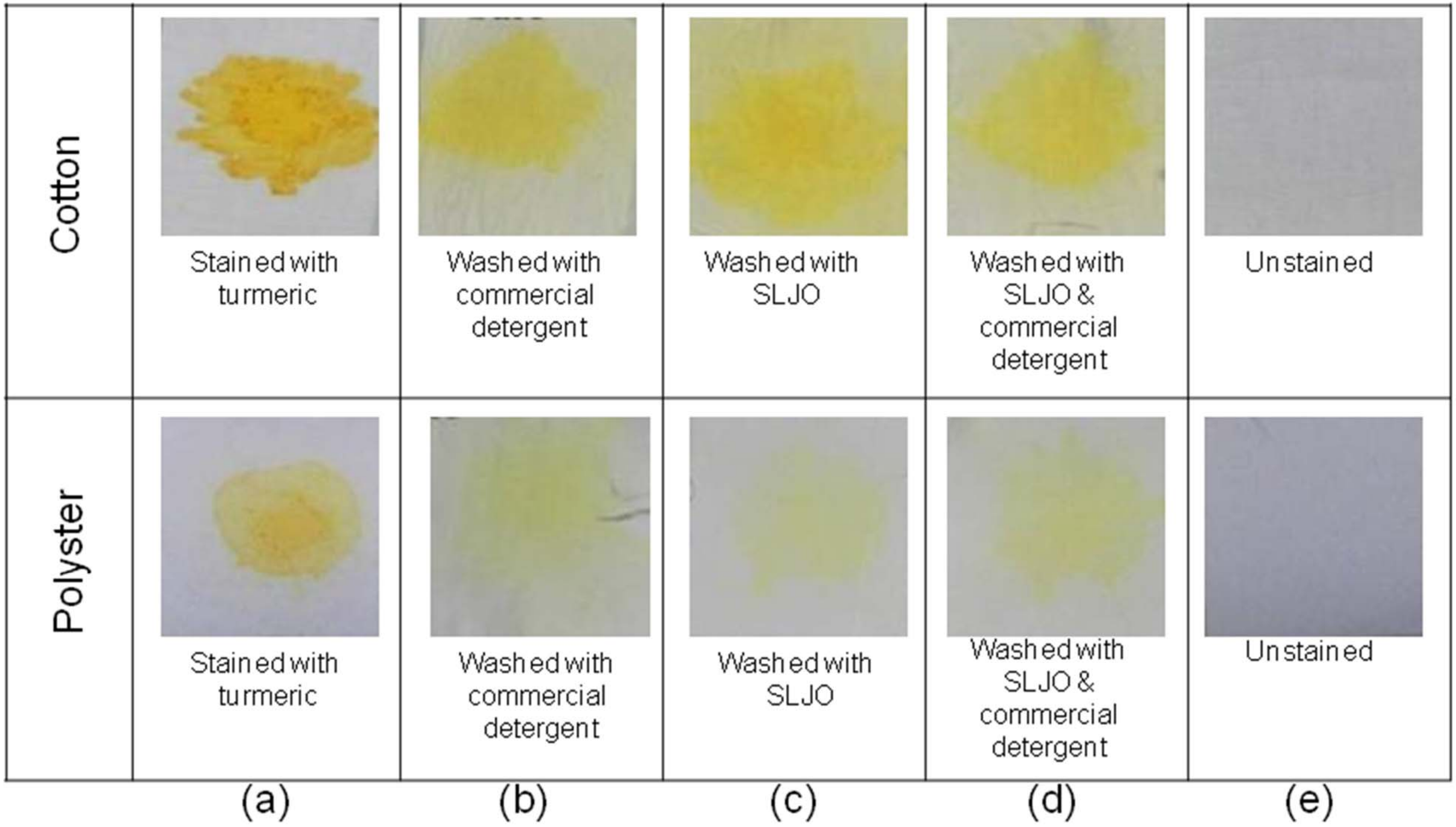

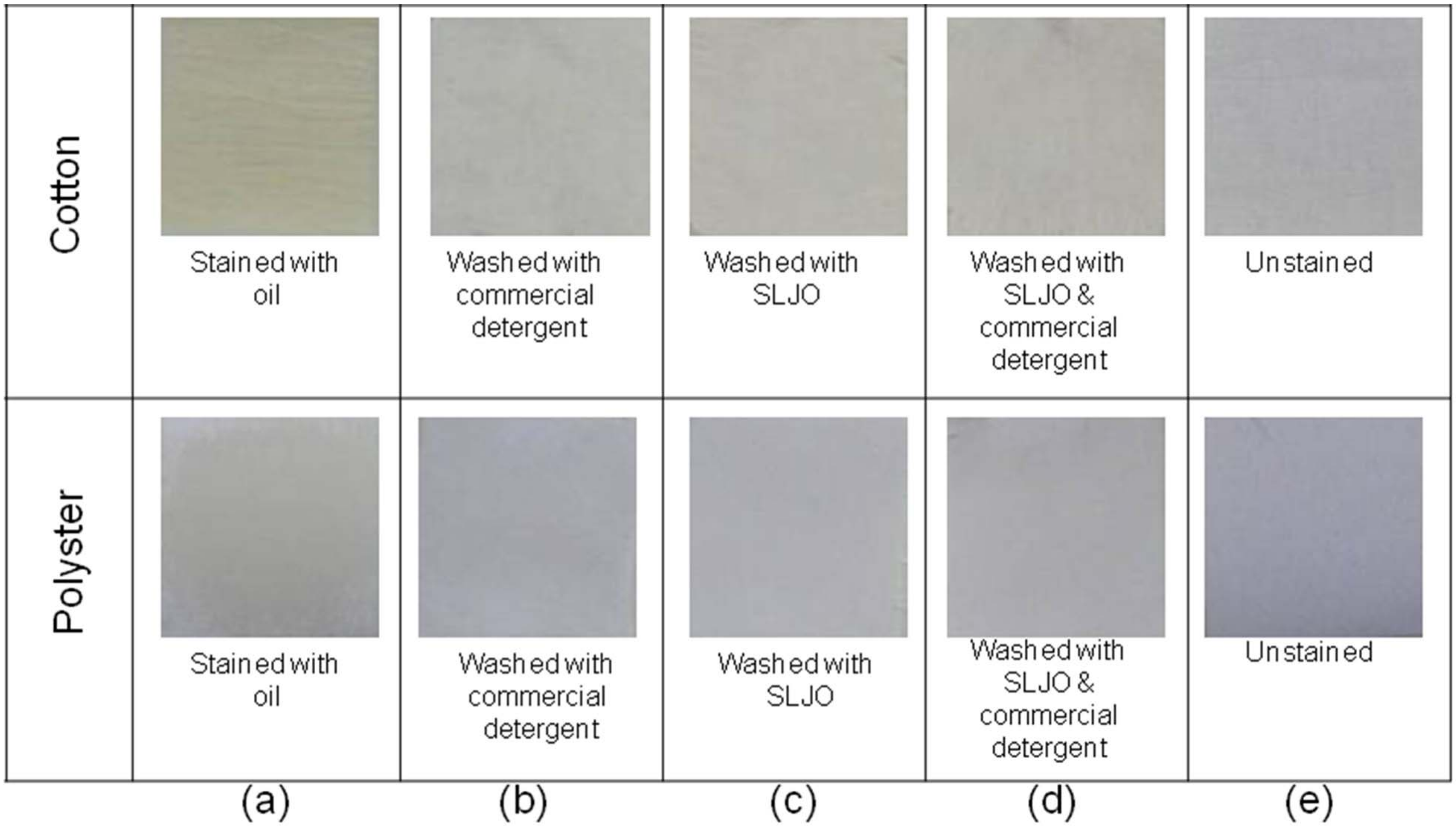
Cotton
Polyster
Stained with oil
Washed with commercial detergent
Washed with SLJO
Washed with SLJO & commercial detergent
Unstained
Stained with oil
Washed with commercial detergent
Washed with SLJO
Washed with SLJO & commercial detergent
Unstained
(a)
(b)
(c)
(d)
(e)

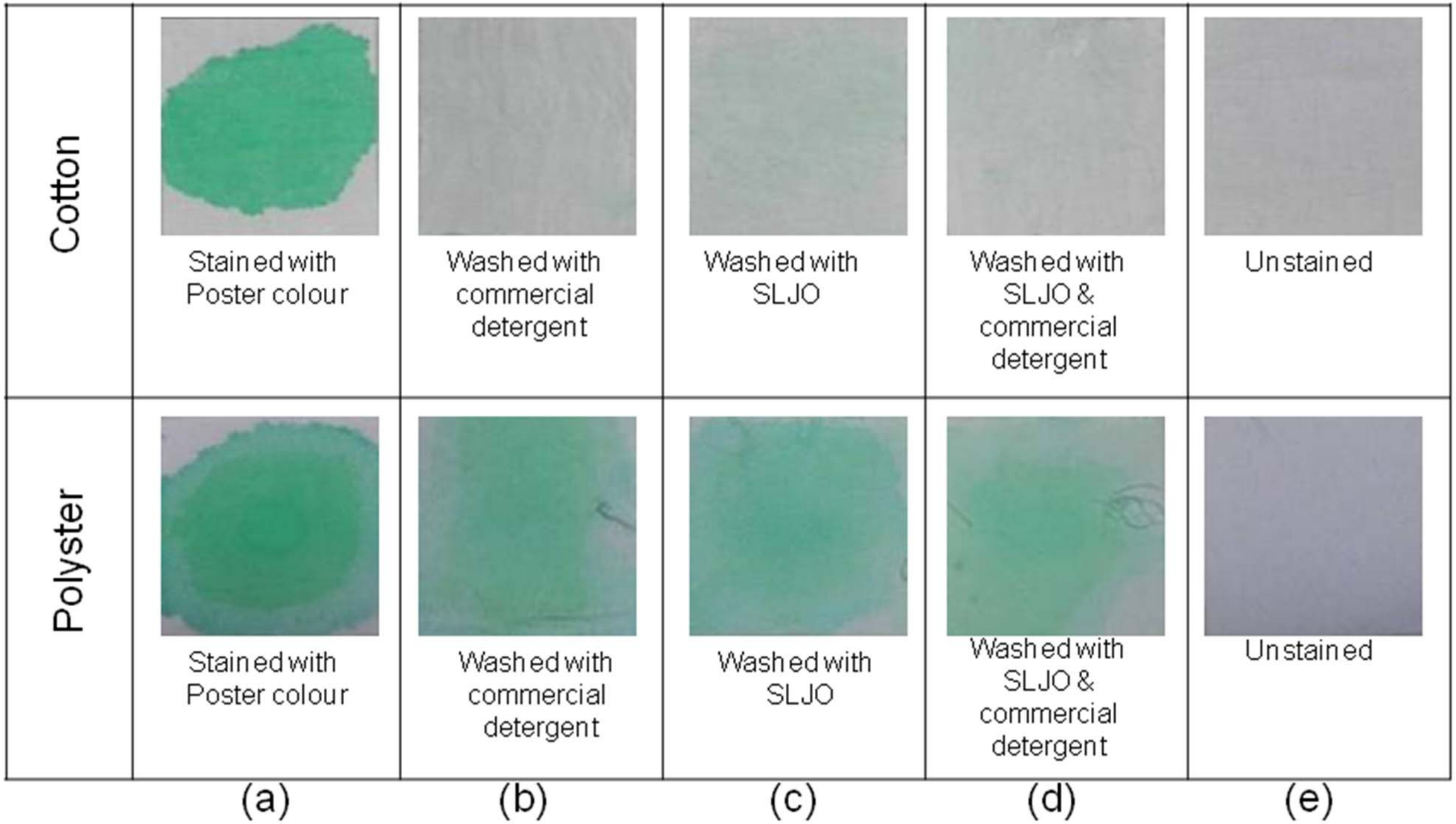
Cotton
Stained with Poster colour
Washed with commercial detergent
Washed with SLJO
Washed with SLJO & commercial detergent
Unstained
Polyster
Stained with Poster colour
Washed with commercial detergent
Washed with SLJO
Washed with SLJO & commercial detergent
Unstained
(a)
(b)
(c)
(d)
(e)
